# Interplay between yielding, ‘recovery’, and strength of yield stress fluids for direct ink writing: new insights from oscillatory rheology†

**DOI:** 10.1039/d4sm00758a

**Published:** 2024-09-11

**Authors:** Rishav Agrawal, Esther García-Tuñón

**Affiliations:** a School of Engineering and Materials Innovation Factory, University of Liverpool UK ragrawal@liverpool.ac.uk egarciat@liverpool.ac.uk

## Abstract

Formulation design and rheology are critical for successful manufacturing *via* direct ink writing (DIW), thus linking rheology and printability is a growing area of research amongst the DIW and rheology communities. This work provides an extensive rheological investigation into the material strength, yielding and ‘recovery’ properties of graphite (Gr)-hydrogel based formulations. Using state-of-the-art Large Amplitude Oscillatory Shear (LAOS) techniques, Fourier Transform (FT) rheology and sequence of physical process (SPP) analysis, and 3-step ‘recovery’ tests we provide new insights on the yielding phenomenon, energy transitions and microstructural changes that the formulations undergo. The insights from the rheology experiments are combined with *in situ* and continuous monitoring during the printing process. From these analyses, we select rheological metrics or descriptors to quantify flowability, recoverability, and material strength. There is a threshold concentration of Gr powders (30 wt%) at which there is a shift in the yielding process. Below this threshold (for the F127 hydrogel and mixtures with low Gr content), perfect plastic dissipation ratio (*ϕ*) values are close to 0 in the LVR and then steeply increase to close to 1 after the cross-over in a narrow strain (and stress) space. As Gr concentration increases, and print quality gets worse, *ϕ* values consistently increase in the LVR and at any given *γ*_0_, evidencing an increased energy dissipation throughout the flow transition region. Lissajous–Bowditch curves and SPP Cole–Cole plots illustrate these trends. The extent of the ‘recovery’ (quantified by the mutation time, *λ*_I_, and the storage modulus ‘recovered’ after large deformations 
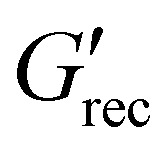
) is also directly related to Gr content, with higher loading resulting in lesser recovery. Our findings provide a comprehensive set of metrics to characterise complex (yield stress) fluids for DIW using three property maps, one for each stage: flowability or yielding process, recoverability and material strength. The results demonstrate that considering these three maps holistically provides insightful trends to guide formulation design and assess performance in DIW.

## Abstract 2

We propose a new framework to characterise yield stress fluids for direct ink writing that enables us to link trends in rheological metrics with printing resolution.

## Graphical abstract

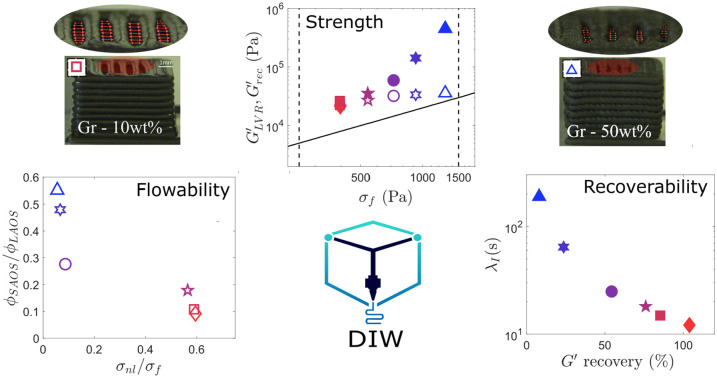

## 1 Introduction

Direct ink writing (DIW) is an extremely versatile additive manufacturing technique used in applications ranging from (but not limited to) batteries,^1^ capacitors,^2^ composites^3^ and bioprinting.^4^ DIW, also known as robocasting, is a three-dimensional (3D) printing technique that involves the continuous extrusion of colloidal pastes or ‘inks’ through a fine nozzle to create 3D structures.^5–7^ These ‘inks’ are complex fluids that generally consist of highly concentrated colloidal suspensions of powders (ceramics, carbon based and 2D materials)^3,6,8^ or hydrogels in bioprinting applications.^9,10^ The formulations often contain the material of interest (‘active’ material) combined with different additives (*e.g.* binders and rheology modifiers). DIW is one of the most versatile additive manufacturing techniques in terms of materials formulation. However although highly exploited in research labs, DIW technology has not yet fully flourished partly due to the highly specialized fundamental understanding required in formulation and rheology of complex fluids. On a more positive perspective, many efforts are currently being invested in this area, as recent research papers and reviews attest.^11–16^ In this work, we delve into the rheological analysis of a representative set of printable samples that exhibit different behaviours during the printing process resulting in printed parts with different qualities. We aim to elucidate the differences between their microstructures and rheological behaviours through a comprehensive set of metrics using oscillatory rheology. DIW formulations are ‘soft solids’ that should also be yield stress and highly shear thinning fluids. They must flow during the extrusion process; be able to recover their original (or at least a minimum) strength quickly after printing; and to retain the pre-designed shape without deformation, while supporting the printed structure.^17^ Yield stress fluids are ubiquitous in many other environmental, biological and industrial applications, *e.g.* muds and soils, soft tissues and drilling fluids. Thus understanding the physics of yielding has been the focus of extensive research, with exciting advances being made and open questions to be answered to better understand variations in failure mechanism as in the field of solid mechanics.^18,19^

Within additive manufacturing, the concept of “printability” and the design of yield-stress fluids highly depends on the application. For example, low stiffness and yield stress values are required to ensure cell viability^10^ in bioprinting; whereas other applications require ‘stiff’ formulations to print structures with very high resolutions. The precise relationship between the rheology and printability of complex fluids for DIW must be considered and quantified in context. Despite the diversity of soft solids being printed for different applications, the systematic rheological studies and guidelines we report here (and in previous work^13,17^) to study formulations for DIW are common to all applications.

From a functional materials perspective, graphite (Gr) formulations have been used for different purposes in DIW, either as active or support material. For example, as active material in stretchable strain sensors and energy applications.^20–22^ Piezoresistive ‘soft’ pressure sensors with very good coefficient of variation for the base conductivity and piezoresistive response have been made using DIW.^23^ Graphite has also remained among the most common anode material used when manufacturing energy storage devices using lithium-ion batteries (LIBs).^24–27^ Although the application of LIBs has been rapidly increasing and is projected to show a steady rising trend, the research on the LIBs manufacturing process is still lacking.^28^ Making electrodes for LIBs include several steps *e.g.* mixing, coating, stacking, and many of these steps involve dealing with the electrode in its ‘slurry form’. Understanding the rheological properties of these slurries will also help in optimizing the manufacturing process thus saving energy and increasing the throughput of LIBs. Graphite inks are also considered in the manufacturing of multi-material structures for energy applications using DIW.^7,29^

Graphite can also be used as temporary support material to aid the additive manufacturing of complex ceramic structures.^30,31^ Once printed, graphite and additives can be easily removed during the sintering of the ceramic part at high temperatures. This must be done at a slow heating rate in air (or oxidizing atmosphere) to facilitate the burn out of the carbon while avoiding damage to the ceramic part. For example, graphite formulations have been used in embedded printing to create micro channels in bulk alumina parts^31^ or as temporary support scaffold to create ceramic spheres.^30^ Embedded printing is an emerging variant of the DIW technique where a complex structure can be printed inside a soft supporting matrix.^32,33^ All these studies showcase the huge potential of graphite in formulations for DIW. It is worth noting, that the rheological needs change in embedded printing,^31^ hence the context is crucial when discussing ‘printability’. Other modified DIW technologies (*e.g.* extrusion coupled with UV curing, or *in situ* heating) are designed to perform “solidification” *via* “*in situ*” post-processing steps.^34^ For these derived technologies, the ‘inks’ rheology will evolve with irradiation, time, temperature or both. The study of evolving inks needs to be adjusted as appropriate, for example by studying how their properties evolve with UV-curing, or temperature. To achieve this, additional metrics and bespoke maps will be required.

For all these applications, it is imperative that the inks have suitable rheological properties so that they flow easily through the printing nozzle, and retain the filament shape in a short timescale after printing. Despite the progress made in DIW research (for Gr inks in particular), there are still very few studies that report a comprehensive and relevant rheological characterization of the (Gr-based) printable formulations. Often, only a brief discussion of a flow or viscosity curve is provided, and some studies do not provide any rheological characterizations at all. More broadly, measuring and reporting the rheology of complex fluids in DIW is a clear area to improve as recent reviews also highlight.^14,15^ This has led to a bottleneck in the widespread use of DIW compared to some other 3D printing techniques such as ink-jet printing or fused deposition modelling.

To address this, here we carry out a thorough analysis of oscillatory measurements to identify rheological metrics that can elucidate the characteristic behaviours that the formulations display during the printing process. Using *in situ*-monitoring enables us to identify macroscopic distortions on printed parts, such as spreading or merging of the filaments, and loss of the pre-designed spacing between lines. Our previous work^13^ and the terminology we use here align with a recent review by Liu *et al.*^14^ on the link between rheology and printability, that considers three critical rheological parameters to define printability: flowability, recoverability and material strength. Flowability refers to the flow (yielding phenomenon) of the material during the extrusion process that has often been studied using flow experiments in continuous shear and simple LAOS analysis. Recoverability refers to the materials ability to quickly regain its original strength after printing to avoid sagging or collapsing of the printed structure.^14,15^ Here the term ‘recovery’ is used in the DIW context; it refers to the ability of the material to rebuild its original structure and ‘recover’ its mechanical strength. This should not be confused with the traditional term that refers to the part of the deformation that is recoverable. To be clear, in this study we have not studied or quantified recoverable and unrecoverable processes which is the goal of future research. DIW ‘recovery’ tests have been previously done to monitor and quantify (using the mutation time, *λ*) the rheological behaviour during step strain change experiments. Instead of oscillatory shear other authors implemented a three interval thixotropy test (3ITT)^12^ using continuous shear at varying rates for hydrogel inks. Material strength, the property most often discussed in DIW, is related to what we refer to as the ‘flow’ stress (quantified at the moduli crossover (*G*′ = *G*′′) that falls in the plateau region in the *σ*_0_*vs. γ*_0_ curves),^13^ and the elastic and viscous moduli (*G*′,*G*′′) in the linear viscoelastic region (LVR).

Previous studies on the rheological behaviour of electrode slurries are based on simple flow curves, or the ‘traditional’ amplitude sweep analysis which is valid for the linear response. There are some but very few studies that delve into non-linear rheology and its link to printing or manufacturing performance. For example, the yielding behaviour of anode slurries (containing Gr as the active material and carbon black (CB) as the conductive additive combined with carboxymethyl cellulose (CMC) as a binder) has been studied.^35^ The authors implemented the sequence of physical processes (SPP) technique to study the intra-cycle rheological transition under oscillatory shear flow.^36–38^ We have recently reported^13^ the rheological behaviour of a different set of carbon-based formulations (containing graphite (Gr), graphene oxide (GO) and carbon nanotubes (CNTs)) using Fourier Transform (FT) rheology. We implemented an existing mathematical framework^39,40^ to identify the onset of non-linearities by examining the appearance of higher (odd) harmonics in the output stress signals obtained during the strain sweep experiments. This was crucial to quantify the extent of the solid-to-liquid transition (between the onset of non-linearities and the moduli crossover). Note that we refer to this strain (or stress) space as the flow transition region throughout this manuscript (quantified by the flow transition index, FTI),^17^ that is linked to the flowability of a formulation. The addition of Gr to Pluronic F127 gel had a significant impact on the onset of nonlinearities, which shifted from a strain amplitude of 2.8% (for F127) to 0.04% (F127-Gr). Material measures of nonlinearities, such as the viscous Chebyshev coefficients showed some distinctive extreme trends for not printable formulations, evidencing the need for further analysis to link rheology and the diverse behaviours detected during the printing process.

In this paper, we carry out a comprehensive rheological study of graphite formulations to investigate their flowability (or yielding process), recoverability, and strength, combined with continuous monitoring during the printing process for each formulation. To quantify flowability we use quantitative material measures from state-of-the-art LAOS studies: the onset of non-linearities, *σ*_nl_ and *γ*_nl_ determined using FT rheology, the plastic dissipation ratio (*ϕ*), the FTI^17^ and the cage modulus (*G*_cage_).^41^ To quantify the recoverability and material strength, that are critical in DIW to ensure good shape fidelity of the printed structures,^42^ we use three step strain change experiments, where the strain amplitude is varied from very small to large and again to a small value, mimicking the extrusion-based printing process.^17^ We quantify the recovery test results using extent of recovery and mutation time (*λ*) calculations.^43^ From these analyses, we select rheological metrics that enable us to thoroughly describe and compare a set of formulations that lead to printed parts with notable differences in resolution. We systematically study the impact of adding graphite to Pluronic F127 gels (with Gr concentrations ranging from 0 wt% to 50 wt%) on the three rheological stages linked to printability. The SPP analysis complements these results by providing insights into how the yielding process systematically evolves with Gr content.

In addition, this paper delivers new Ashby-type maps using rheological metrics to represent each of the three stages in the printing process: flowability, recoverability, and strength. These maps should be considered holistically to characterise the properties that can be linked with performance of DIW formulations during printing. Noting that any small change (*e.g.* solid loading) in the formulation can lead to dramatic changes on one or more rheological metrics, and as a consequence in the formulations' printability. We also critically discuss the importance of compromising the printing performance with post-processing or post-printing considerations such as drying, binder removal and functional performance.

## 2 Materials and methods

### 2.1 Graphite powder characterization

**Fig. 1:**
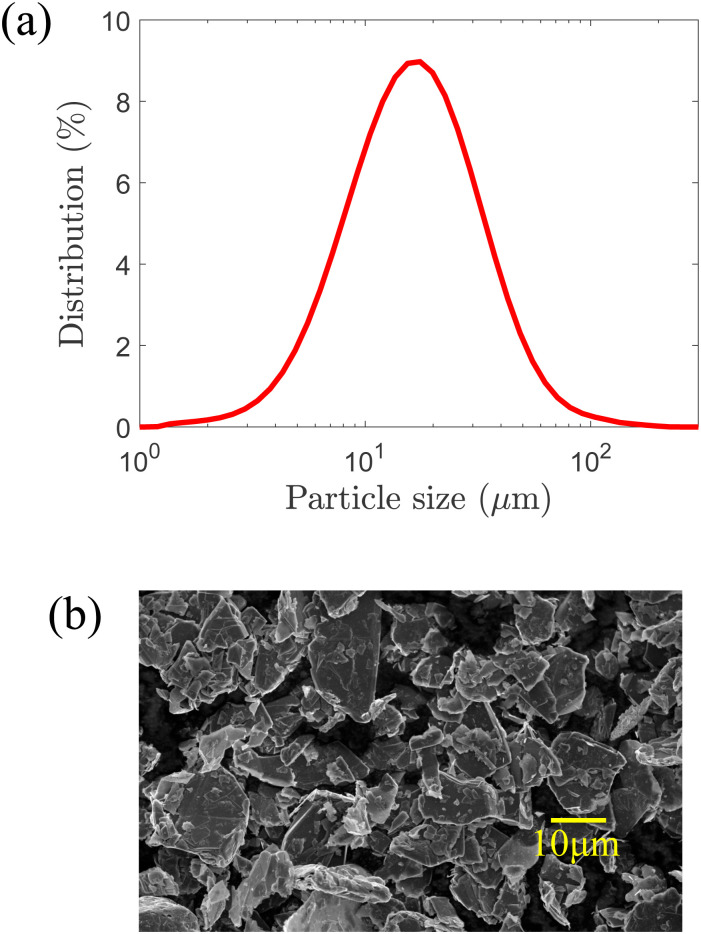
Gr powder characterization results. (a) Particle size distribution measured using DLS. (b) SEM image illustrating the morphology of Gr particles.

The graphite (Gr) powder used is Sigma Aldrich 99% carbon basis Graphite flakes (CAS number: 7782-42-5). Gr powder is sieved to below 100 μm to remove large agglomerates. Gr powder is characterized using dynamic light scattering (DLS) and scanning electron microscope (SEM). DLS is a non-invasive technique that is used to measure the size distribution of particles dispersed in a liquid medium and works on the principle of Brownian motion of dispersed particles.^44^ Gr powder is dispersed in ethanol (the liquid medium), and the DLS measurements are carried out using a Malvern Mastersizer 3000. Measurements are made over a period of 10 s providing a large number of data in order to obtain a good representation of the size distribution of Gr powder, as shown in Fig. 1(a). Gr powder has a wide range of size distribution, varying between ∼1–100 μm. SEM images are taken on a Hitachi S-4800 SEM. Gr particles are stuck to an aluminium stub with an adhesive carbon tab. Samples are then sputtered in conductive chromium for 90 seconds with Q150T Plus sputter coater. The SEM image of Gr particles is shown in Fig. 1(b).

### 2.2 Graphite/pluronic F-127 formulations

We use BASF Pluronic F127 (referred to as F127) powder (purchased from Sigma-Aldrich), BioReagent, as the formulation base. F127 is a triblock copolymer of poly-propylene oxide (PPO) flanked by two poly-ethylene oxide blocks (PEO).^45,46^ It is a nonionic amphiphilic surfactant and in aqueous solutions, has a reversible thermogelling behaviour.^47^ It behaves as a liquid at low temperature (*i.e.* below its critical gelling temperature, CGT), and as a gel above the CGT, making it a convenient formulation base to add and mix other materials (*e.g.* powders). F127 becomes a printable hydrogel above the CGT, which is ∼18 °C at a concentration of 25 wt% in water.^6,29^ F127 has been widely used as the formulation base in DIW because of its versatility to act as an ‘excellent carrier’ of powders.^3,4,6,9,29^

F127 stock solution is prepared by adding F127 powder into distilled water. After a small amount of powder is added to the stock solution, the container is sealed and mixed for 2 minutes in a THIARE250 planetary mixer at 2000 rpm and then defoamed for 2 minutes at 2000 rpm to remove air bubbles. After each mixing-defoaming cycle, the formulation base is cooled in an ice bath to cool the Pluronic down and prevent excess heat build up that can occur after successive mixing cycles. The process is repeated until all powder is added and a 25 wt% suspension had been created which resulted in a soft, transparent gel at room temperature.

Sieved Gr powders are then added gradually to the F127 stock solution in a similar procedure as mentioned above. Small amounts of powder are added gradually followed by a 2 minute mixing-defoaming cycle at 2000 rpm (2 minutes each) in the planetary mixer followed by a few minutes in an ice bath to cool the suspension down to liquid state. The composition of the six different graphite-Pluronic (Gr/F127) formulations that are formulated and used in this study is shown in Table 1.

**Table 1:** Table of Gr-F127 formulations used in this work

Base	Gr content
F127 (25 wt% in water)	Gr – 0 wt%
	Gr – 10 wt%
	Gr – 20 wt%
	Gr – 30 wt%
	Gr – 40 wt%
	Gr – 50 wt%

### 2.3 Rheological measurements

A strain controlled rheometer ARES G2 (TA Instruments) has been used for the rheological measurements. All the measurements are carried out using a 2 mm gap and 40 mm stainless steel sandblasted parallel plate with a solvent trap. The temperature is maintained at 23 °C using a Peltier plate. In order to prevent samples drying over time for the recovery tests (some of which lasted over 30 minutes), a thin layer of low viscosity oil is kept at the edge of the sample.

#### 2.3.1 LAOS measurements

LAOS measurements are carried out in the form of strain amplitude sweep tests. In this test, we apply an oscillatory input strain and measure the resultant output stress for every prescribed strain amplitude. The strain amplitude values (*γ*_0_) ranging between 0.01% and 500% are chosen to investigate the structure deformation from small amplitude oscillatory shear (SAOS) to LAOS, which are commonly carried out in the frequency range between 0.1 and 1 Hz.^6,8,17,48,49^ In this work, the oscillation frequency is kept at 0.5 Hz for all the oscillatory measurements. The ARES G2 rheometer and the TRIOS software allow to collect the strain amplitude sweep data in either correlation mode or transient mode. The former provides the first-harmonic moduli, *G*′,*G*′′ and the latter provides the raw strain/stress waveforms during a strain amplitude sweep. FT-rheology is used to calculate the first-harmonics of the Fourier-transformed stress response, which gives the *G*′ and *G*′′ values. These are traditionally known as storage and loss moduli, respectively. To validate our results, we routinely compare the *G*′, *G*′′ obtained from the FT-rheology analyses of raw waveforms with those provided by the TRIOS software when using the correlation mode. The transient data are collected for 3 cycles of oscillation for every strain amplitude at a frequency of 1024 points per cycle. The LAOS analysis is carried out using the existing mathematical frameworks: Fourier-Transform (FT) rheology, Lissajous–Bowditch (LB) curves, apparent cage modulus (*G*_cage_), plastic dissipation ratio (*ϕ*) and sequence of physical processes (SPP).

#### 2.3.2 Fourier-transform (FT) rheology

For a sinusoidal strain input *γ*(*t*) = *γ*_0_ sin(*ωt*), the stress response can be represented by a Fourier series:^50,51^1
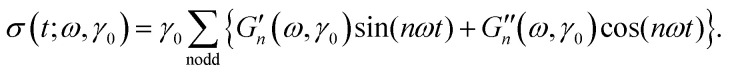


In the linear viscoelastic regime (LVR), the stress response includes only the first-harmonic, *n* = 1. Based on symmetry arguments, the stress response that remains unchanged if the coordinate system is reversed, will result in only odd harmonics in the Fourier series representation.^52,53^ Even harmonics are commonly related to nonperiodic or asymmetric slip/yield responses.^53^

#### 2.3.3 Lissajous–Bowditch (LB) curves, apparent cage modulus and plastic dissipation ratio

In a LAOS experiment, the materials instantaneous properties over the entire oscillation can be visualized using the elastic and viscous LB projections. The shape of the elastic LB curves provides visual information about how the material responds during an amplitude sweep. At small strain amplitudes, a straight line corresponds to purely elastic and a circle to purely viscous; while a rectangle shape at large strain amplitudes corresponds to a perfect plastic behaviour. The distortion of LB curves evidence the raising of non-linear behaviours, while the area within the curve represents the energy being dissipated. The visual information provided by these curves is quantified using the following metrics. The cage modulus, *G*_cage_,^54^ is calculated from the elastic LB curves as the instantaneous slope at zero stress. This is a parameter specific to yield stress fluids to represent the ‘cage’ elasticity (or average energy stored elastically normalised by the recoverable strain amplitude).^54^2
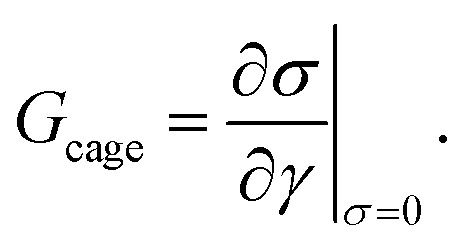


The plastic dissipation ratio, *ϕ* is the ratio of the energy dissipated in a single oscillation cycle (area enclosed in a elastic LB curve, *E*_d_) to the energy that would be dissipated in a perfect plastic response (which would correspond to a rectangle-shape in the elastic LB curves) with equivalent strain amplitude and maximum stress ((*E*_d_)_pp_),^53^3
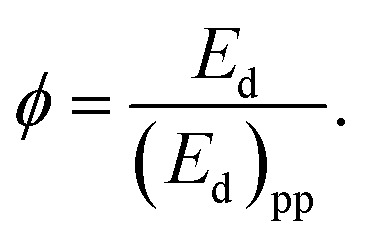


A perfect plastic behaviour corresponds to *ϕ* = 1 and a purely elastic response to *ϕ* = 0.

#### 2.3.4 Sequence of physical processes (SPP)

The sequence of physical processes (SPP) approach has been developed by Rogers and co-workers^36–38,41^ providing new insights on the time dependency of the non-linear rheological behaviours. This technique provides quantitative information for all strain, strain rate, and stress points along the LB curves. It utilizes the Frenet–Serret theorem,^55^ which defines the motion of a moving particle along a curvature with three orthonormal set of vectors direction: the tangent vector (**T**(*t*)) pointing in the direction of the motion, the normal vector (**N**(*t*)) pointing to the centre of the curvature of the motion, and the binormal vector (**B**(*t*)) pointing normal to both **T**(*t*) and **N**(*t*). Each point throughout the LB curve is given by the position vector **P**(*t*) = [*γ*(*t*),*γ̇*;(*t*),*σ*(*t*)], and therefore the three orthonormal vectors are given by,4
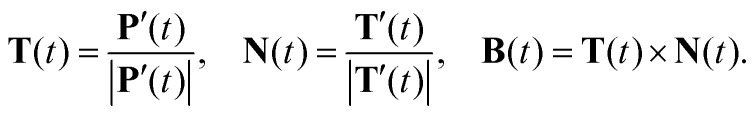


The projections of the binormal vector **B**(*t*) = (*B*_*γ*_,*B*_*γ̇*/*ω*_,*B*_*σ*_) and the orientation of the osculating plane at every point in the LB curves are then used to calculate the transient elastic modulus (
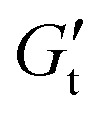
) which quantifies change in stress w.r.t. strain, and transient viscous modulus (
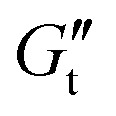
) which quantifies change in stress w.r.t. strain rate,^56,57^5
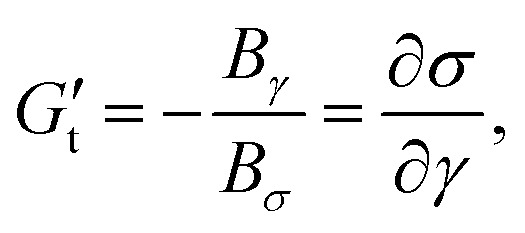
6
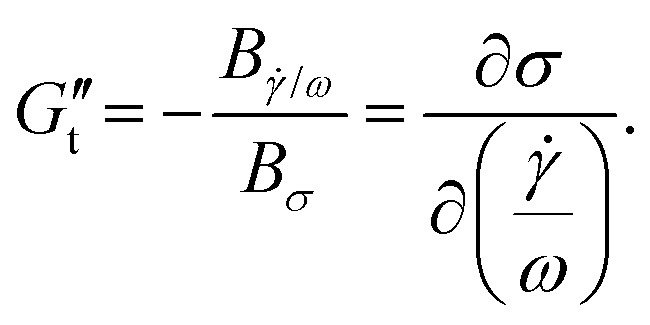


If 
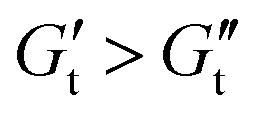
, the material behaviour is predominantly elastic or ‘solid-like’, and if 
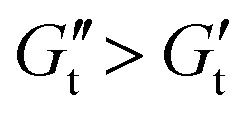
 the material behaviour is predominantly viscous or ‘liquid-like’.

In the SPP analysis, the rheological transition within an oscillation cycle is generally investigated using a transient Cole–Cole plot.^35,37,57–59^ The Cole–Cole plot can be viewed as a trajectory of a point with coordinates 
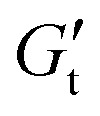
 (transient elastic modulus) and 
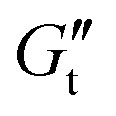
 (transient viscous modulus) in the horizontal and vertical directions, respectively. By examining the trace of 
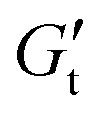
 and 
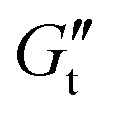
 in the Cole–Cole plot, one may determine the rheological transition in a particular range of interest.^37^ Strain softening or hardening is associated with sideways movements to the left or right, respectively. Shear thinning or thickening is associated with motion to the top or bottom, respectively. The location of the material response in the Cole–Cole plot, [
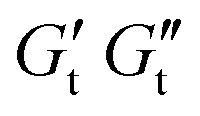
] space, also provides additional information. 
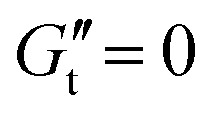
 corresponds to a purely elastic response, whereas 
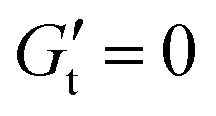
 corresponds to a purely viscous response. When 
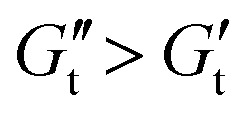
, a response would be said to be predominantly viscous, while 
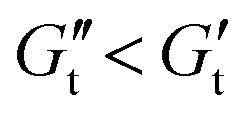
 would be described as a predominantly elastic response. A transition from a predominantly elastic to a predominantly viscous response indicates fluidization, whereas from a predominantly viscous to a predominantly elastic response indicates solidification. 
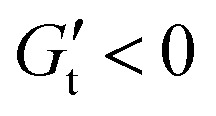
 represents a recoil process, whereas 
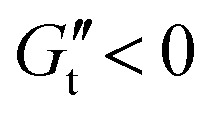
 represents backflow of material.^37^ The raw waveforms data *i.e. σ*(*t*), *γ*(*t*) and *γ̇*(*t*) are analyzed using SPP freeware for MATLAB provided by its developers (https://publish.illinois.edu/rogerssoftmatter/freeware/). The SPP analysis is carried out in one full-cycle using Fourier domain filtering, where first 19 harmonics are used to reconstruct the stress waveform.

#### 2.3.5 Recovery tests

**Fig. 2:**
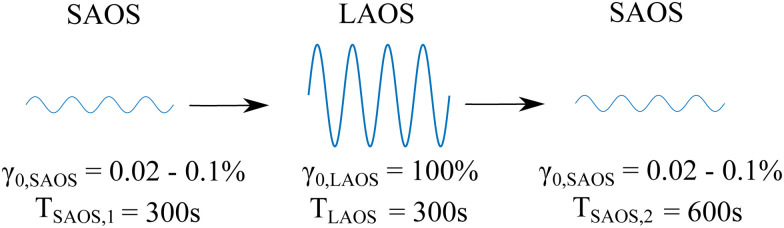
3-step recovery test carried out in this study. The samples are subjected to an initial small amplitude strain for 300 s, followed by large amplitude strain to ensure that they are in the ‘liquid-like’ regime, followed by another 600 s of small amplitude strain to monitor the recovery of the moduli. The oscillation frequency is kept constant at 0.5 Hz. For Gr – 0 wt% and Gr – 10 wt%, *γ*_0,SAOS_ is 0.1%, for Gr – 20 wt% and Gr – 30 wt%, *γ*_0,SAOS_ is 0.05%, and for Gr – 40 wt% and Gr – 50 wt%, *γ*_0,SAOS_ is 0.02%.

Here the term ‘recovery’ should be considered in the DIW context, understood as the ability of the material to rebuild its original structure (and ‘recover’ its mechanical strength)^14,17^ when exiting the nozzle tip to maintain a high resolution of the printed structures. A three step ‘recovery’ test is carried out to monitor the reformation of the material's structure by shearing the material from SAOS to LAOS to SAOS.^17^ A constant strain amplitude of *γ*_0,LAOS_ = 100% is applied to all the formulations in the LAOS region. The strain amplitude in the SAOS region (*γ*_0,SAOS_) is varied such that it remains in the LVR for each sample, as there exists a dependence of the end of LVR (denoted by *γ*_nl_) with increasing Gr concentrations (discussed in Section 3.3). The duration of the first SAOS interval is 300 s to monitor the initial strength of the material. The LAOS interval for 300 s ensures the material reaches steady state. The third step (SAOS) for 600 s monitors the recovery over a longer period of time to study the timescales and extent of the recovery. This protocol is summarized in Fig. 2.

The recoverability is quantified by the extent of *G*′ recovery, calculated as the *G*′ percentage between the first and second SAOS steps: 
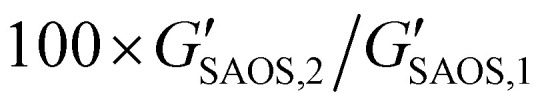
. The recovery timescale for each formulation is quantified using the ‘mutation time’ (*λ*).^17,43,60^*λ* scales the magnitude of the gradients of the recovery with the final stiffness (
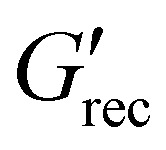
) of the network, and is given by7
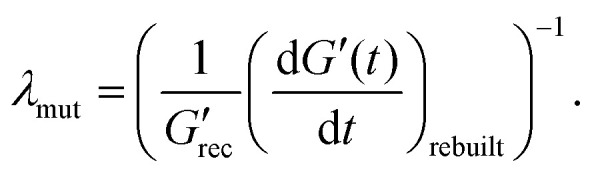


### 2.4 Direct ink writing

**Fig. 3:**
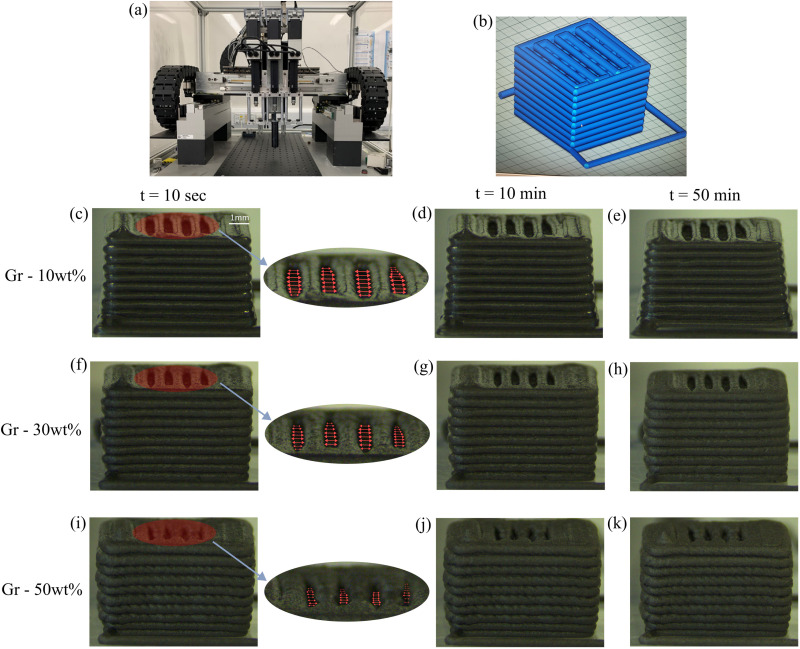
(a) Robocaster used in this work with loaded syringe inside black collar in the centre. (b) CAD image of the desired 3D structure with a “lead-in” line. 3D printed structures of Gr formulations at concentrations of (c), (d), (e) 10 wt%, (f), (g), (h) 30 wt%, and (i), (j), (k) 50 wt%. Time evolution of the structures is studied where (c), (f), (i), (d), (g), (j), and (e), (h), (k) show the structures 10 seconds, 10 minutes and 50 minutes after printing, respectively. The resolution of the spacing between filaments in the top layer considerable drops as Gr content increases (shown as highlighted regions in (c), (f), (i)).

DIW of the formulations is carried out using a custom built robocaster^6,29^ (shown in Fig. 3(a)). It has three individual syringe plungers allowing it for multi-material printing. These plungers are powered using an additional set of linear actuators that helps in depositing the printing inks. This robocaster is controlled by an Aerotech A3200 machine controller using G-code. Formulations (Table 1) are loaded into a 5 ml syringe (Nordson, EFD) using a spatula by carefully spreading them over the mouth of the syringe in order to ensure no air bubbles are trapped inside. The material is then pressed down the barrel of the syringe using a custom built plunger until it is near the end of the syringe. The stainless steel (SS) nozzle tips are then screwed on the end of the syringe with a luer lock ready for printing. Desired 3D printed parts are created using the RoboCad software. The structure used in this study (as shown in Fig. 3(b)) has a theoretical dimensions of 5 mm by 5 mm with 10 vertical layers stacked on top of each other. A dummy line (also known as “lead-in” of approximately 25 mm) is printed immediately before the start of each part to ensure flow is steady and stable as the part is printed. After the shape has been created by the user, the RoboCad software splits the shape up into a number of layers. It then generates the G-code and imports this into the Aerotech motion composer. The parts are printed straight onto polytetrafluoroethylene (PTFE) substrates. The printing parameters such as the nozzle tip diameter, printing speed and extrusion rate can influence the print quality.^27^ Here, the same tip diameter *i.e.* 0.51 mm and printing settings are used for all formulations. Once a printed part is completed, it is left to dry at room temperature ≈22 °C and kept under examination for around 1 hour. The printing process and evolution of the printed structure over time are recorded using a Basler acA1920-155uc USB 3.0 camera. It has a Sony IMX174 CMOS sensor and can record at 164 frames per second at 2.3 MP resolution. THORLABS 12X zoom lens is used in conjunction with the camera for the visualization. The recordings are made at 2 frames per second for one hour period in order to examine the structure evolution over time.

## 3 Results and discussion

### 3.1 Direct ink writing of graphite-hydrogel formulations

All the formulations in this work are ‘printable’. They flow through the nozzle tip with ease (no noticeable disruptions in the flow); they recover to different extents when shear forces cease as they are deposited on the printed platform; and they reproduce (to different extents) the pre-designed 3D shape (Fig. 3). However, the quality of final prints depends on the formulation, or Gr content (Fig. 3). Continuous monitoring *in situ* enables us to assess the quality or resolution of each print by comparing the morphological features (*e.g.* filament shape, spacing between filaments and layers, and part dimensions) of each print with those of the original design (Fig. 3(b)). Monitoring is also used to inspect the overall shrinkage of the parts over time. The videos showing the printing of Gr formulations can be found in the ESI.† Immediately after printing, the parts made using formulations with higher Gr content (30 and 50 wt%) showed worse resolution or shape retention (Fig. 3(f) and (i)) than the sample with only 10 wt% (Fig. 3(c)). Note the features in the top layer for each sample (Fig. 3(c), (f) and (i)); there is an obvious loss of resolution in the gaps between the filaments. At low Gr concentrations (Fig. 3(c)) the printed part is a good replica of the CAD design (Fig. 3(b)), with good definition of the pre-designed spacings. However, these morphological details are lost as the Gr concentration in the formulation increases. In order to establish a quantitative comparison of print “quality”, the area in between filaments (top layer) was compared through image analysis (Fig. 3). The normalised area is reduced from 100% (sample with 10 wt% Gr) down to 79% and 48% for the samples with 30 and 50 wt% Gr respectively, confirming the obvious distortion of the printed part as Gr content increases. The desired macro-porosity is not achieved at the highest solid loading (Fig. 3(i)); the printed part shows overall poor shape fidelity. This trend evidences that increasing the Gr content does have an impact on the structural recovery of the filament, leading to a loss of resolution in the final part. The quantification of the recoverability of the inks at different Gr concentrations is extensively discussed in Section 3.4.

We could be misled to consider that the formulation with 10 wt% Gr is the ‘best’, however, in DIW the post-processing steps are as critical as the formulation and printing stages. In colloidal processing every stage of the process has an impact on the quality and performance of the final part.^61^ The post-processing stages will depend on the material used, for example, ceramics commonly go through drying and sintering stages. The drying of ceramic parts is a critical stage to avoid defects that may result in poor mechanical performance. The sintering conditions (oxidizing or reductive atmosphere, heating rate, maximum temperature, *etc.*) will be specific to each ceramic and formulation additive.^62^ For carbon-based materials, each will go through different post-processing steps, for example, graphene oxide printed parts will require freezing before thermal annealing.^8,62^ A systematic study of post-processing conditions for Gr printed parts and their functional performance is not the scope of this work, however, what is common to most post-processing steps in DIW is the drying stage.

The results show that after only 50 min drying in air, the shape fidelity of the printed part drops for the formulation with 10 wt% Gr (Fig. 3(e)). Uneven drying at the top and bottom leads to distortions and uneven shrinkage of the structure. Whereas no such shrinkage of the structures over time is seen for the part printed with the 50 wt% Gr formulation (Fig. 3(k)). This is why formulations with high solid loading, low water and additive content are best from a materials processing perspective. It is therefore important to compromise between the shape fidelity of the filaments and the shrinkage of the structures over time, and more broadly with any post-processing stages that the parts might undergo. It should be noted that the drying steps might be irrelevant in bioprinting applications with hydrogels containing living cells. In the next sections we analyse the rheological behaviour of all formulations and discuss the observed trends with increasing Gr concentrations and the loss of printing accuracy. However post-processing considerations will always be important and specific to each material and application.

### 3.2 Stress–strain curves and first-harmonic moduli

**Fig. 4:**
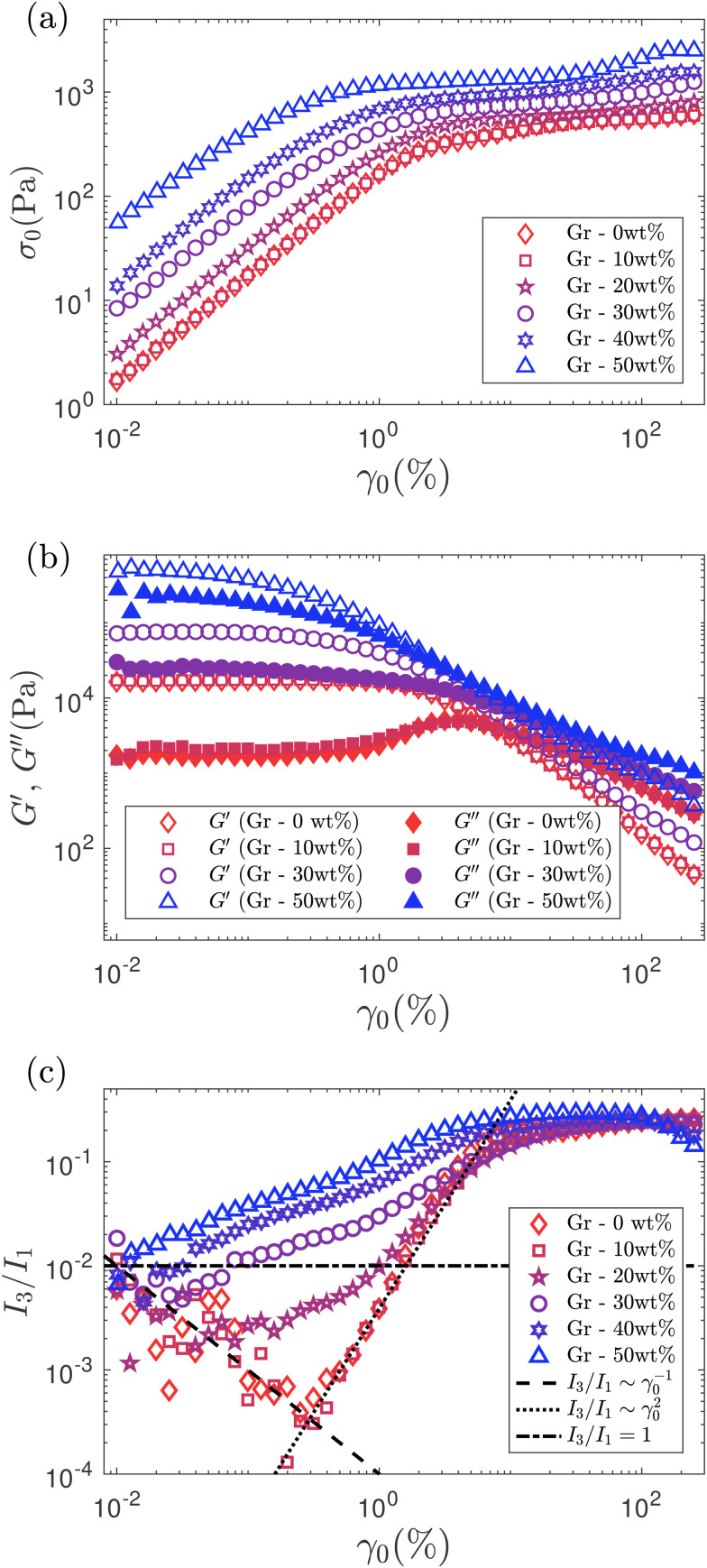
Oscillatory strain sweep test results: (a) Stress *vs.* strain amplitudes showing a linear relationship al low *γ*_0_ and a plateau region at higher *γ*_0_, (b) *G*′ and *G*′′ *vs.* strain amplitudes. (c) Normalized intensities of third (*I*_3_/*I*_1_) harmonics as a function of strain amplitude (*γ*_0_,%). Black dashed line indicates *I*_3_/*I*_1_ ∼ *γ*_0_^−1^ and black dotted lines indicates *I*_3_/*I*_1_ ∼ *γ*_0_^2^. Horizontal dashed dotted line show the threshold for the onset of nonlinearities (*I*_3_/*I*_1_ = 0.01) used in this work. The *I*_3_/*I*_1_ data at very low strains (≈0.01%) for low Gr concentrations are treated as instrument noise and therefore are ignored. All oscillatory data are acquired at a fixed oscillation frequency of 0.5 Hz.

The onset of non-linearities which marks the start of the solid-to-liquid transition (that we refer to as flow transition region) is determined using FT rheology of stress waveforms. This is the point at which the response of the material starts to change (perhaps due to softening, or local re-arrangements with the onset of yielding) at the end of the so called LVR (discussed in Section 3.3.1). At small strain amplitudes, the stress *vs.* strain amplitude variations for Gr formulations show a linear trend (Fig. 4(a)). As the strain amplitude increases beyond the LVR, all the samples show a smooth transition to a ‘plateau’ region around the critical strain amplitude (*γ*_f_). None of the samples show maximum values that could flag any potential issue with stress build-up during the solid-to-liquid transition.^13^*G*′ and *G*′′ values increase with increasing Gr concentrations at lower *γ*_0_ values (Fig. 4(b)). The moduli crossover at the critical strain (*γ*_f_) falls within the ‘bulk’ flow plateau (when *G*′ = *G*′′) that we quantify using the critical bulk flow strain (*γ*_f_) and stress (*σ*_f_). We refer to “flow” stress, *σ*_f_ (which is sometimes considered as the yield point in literature), to mark the end of the flow transition region and calculate the FTI.

### 3.3 ‘Flowability’: yielding behaviour

Determining the yield point is one of the primary goals to characterize yield stress fluids. However, there remains a lack of agreement on the best technique to determine the yield ‘point’.^63^ In the field of solid mechanics, there is a clear difference between brittle and ductile behaviours. Brittle solids (*e.g.* ceramics) are strong but not tough, this is because they do not yield, they break catastrophically. Ductile materials deform elastically at small strains with a linear stress–strain behaviour; yield when this relationship becomes non-linear; and then continue to deform plastically until they break (at the rupture point). The differences between these behaviours lie in the fracture mechanisms, crack origin and propagation.^64^ Many solid materials display mechanical properties (*e.g.* strength and toughness) that are often mutually exclusive.^64^ Researchers in this field devote many efforts to the design of composites inspired by nature (*e.g.* nacre) to achieve both, strength and toughness simultaneously.^65^ The characterisation of mechanical properties of ductile solids (*e.g.* plastics) is standardised, the yield point is understood as the onset (start) of the yielding process which ends at the rupture point. In the yield stress fluids field, yielding is also understood as a process,^18,41^ with recent progress to elucidate brittle and ductile behaviours in soft matter, thus providing the understanding and evidence that not all yielding is the same.^18,19^ However, there are not established standards to quantify this process yet (to the best of our knowledge), nor unanimous agreement within the field. Several techniques based on both continuous and oscillatory shear experiments have been reported, however each of these techniques has its own challenges and uncertainties in the determination of yield stress.^66^ Using steady-state flow curves, the stress data can be extrapolated to the zero shear rate using different rheological models such as the Herschel and Bulkley model^67^ to obtain the yield stress. For oscillatory amplitude sweeps, the crossover between *G*′ and *G*′′ or the point where *G*′ starts to drop below 5% or 10% of *G*_LVR_ value are most commonly used to define yield stress. In this manuscript we describe and compare DIW formulations based on the ‘flow transition’ region as explained in the introduction and Section 3.2.

#### 3.3.1 Onset of non-linearities and yielding process

**Fig. 5:**
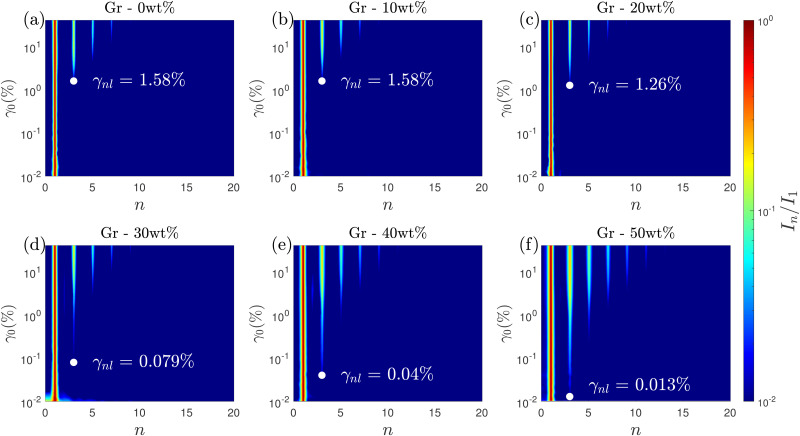
2D projection of normalized stress harmonics (*I*_*n*_/*I*_1_) as a function of the strain (*γ*_0_,%) and multiples of the fundamental frequency (*n*) for Gr concentrations of (a) 0 wt%, (b) 10 wt%, (c) 20 wt%, (d) 30 wt%, (e) 40 wt% and (f) 50 wt%. The white dots and the *γ*_nl_ show the onset of nonlinearities (*I*_3_/*I*_1_ > 0.01) for each sample. The harmonics (*I*_*n*_/*I*_1_) appearing as streaks with red/yellow in the center and blue on the edges is due to the face interpolation scheme used for plotting these maps.

**Fig. 6:**
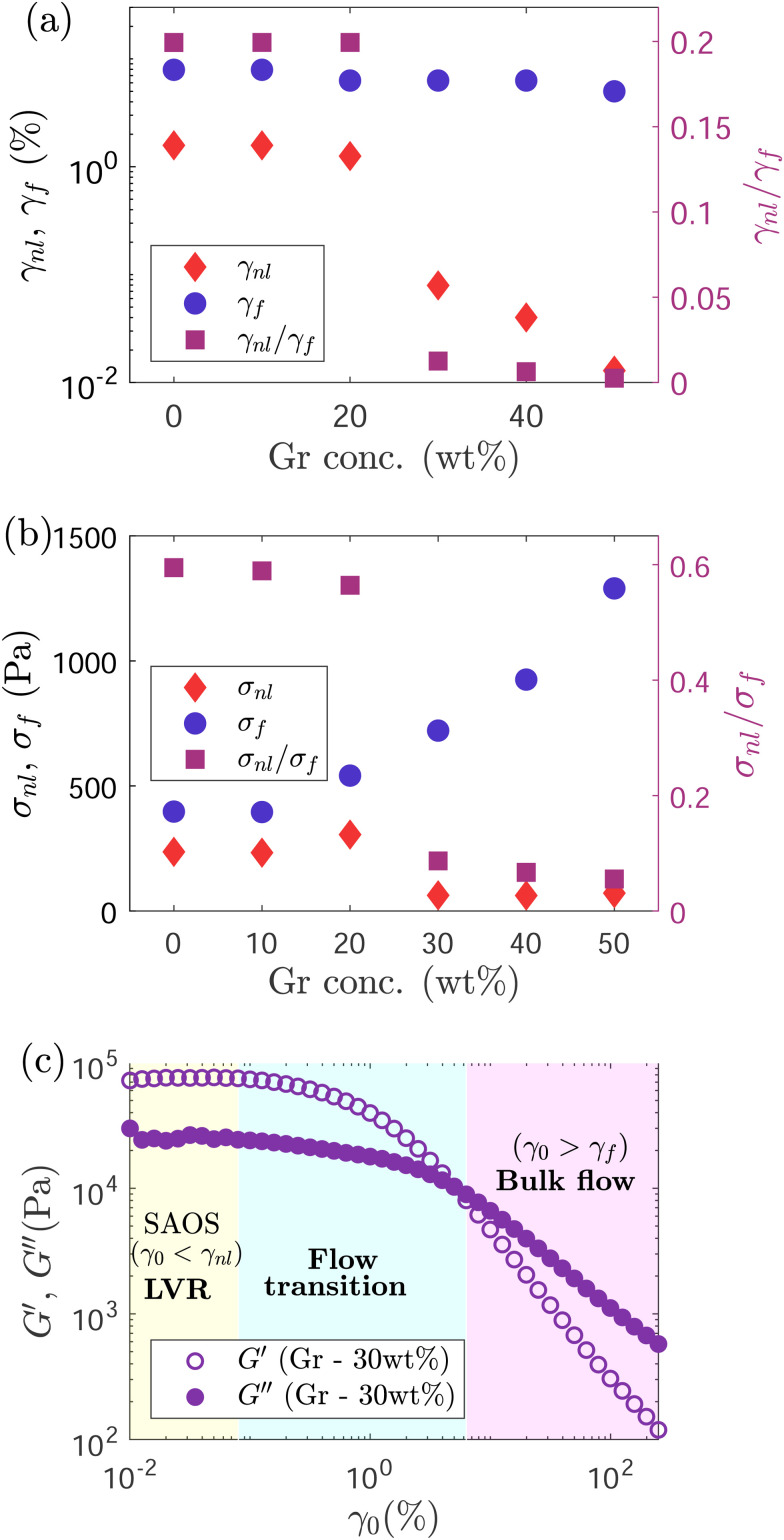
(a) Evolution of yield strain (*γ*_nl_, determined based on the appearance of third harmonic in the stress spectra), flow strain (*γ*_f_, calculated based on when *G*′ = *G*′′), and *γ*_nl_/*γ*_f_ and (b) stress at onset of non-linearities (*σ*_nl_), flow stress (*σ*_f_) and *σ*_nl_/*σ*_f_ with increasing Gr concentrations. (c) Strain amplitude sweep result for Gr – 30 wt%, highlighting the LVR, flow transition and bulk flow regions. In this work SAOS, flow transition and bulk flow regions correspond to LVR (*γ*_0_ < *γ*_nl_), *γ*_nl_ < *γ*_0_ < *γ*_f_ and *γ*_0_ > *γ*_f_, respectively.

FT rheology provides a quantitative measure of the onset of non-linearities that we consider as the start of microstructural changes *e.g.* softening or local yielding. This is carried out based on the appearance of the first non-linear harmonic *i.e.* third-harmonic of the oscillation stress.^13^ We investigate the evolution of *I*_3_/*I*_1_ with increasing *γ*_0_ for different Gr concentrations (Fig. 4(c)). For Gr concentrations ≤10 wt%, *I*_3_/*I*_1_ ∼ *γ*_0_^−1^ at small strains, indicative of the instrument noise, and *I*_3_/*I*_1_ ∼ *γ*_0_^2^ at strains up to *γ*_0_ ≈ 5%, indicative of the orientation and stretch of the polymer chains.^68,69^ At higher solid loading, we get away from this “ideal” behaviour generally observed for polymeric systems,^70^ and for Gr – 50 wt%, no such scaling for the third harmonic intensity is seen (Fig. 4(c)). The onset of non-linear behaviour is determined when the intensity of the third-harmonic (*I*_3_) is greater than 1% of the first-harmonic (*I*_1_)^13^ (Fig. 5). The non-linearities start at similar strains (called *γ*_nl_) for Gr concentrations up to 20 wt%, and then there is a continuous decrease in the *γ*_nl_ values as we approach Gr concentrations of 50 wt% (Fig. 6(a)). *σ*_nl_ remains between 200 and 400 Pa for solid loadings ≤20 wt% Gr; it then decreases sharply below 100 Pa for solid loadings ≥ 30 wt% Gr (Fig. 6(b)).

The onset of non-linearities marks the end of the LVR (at *γ*_0_ > *γ*_nl_) and the start of the solid-to-liquid transition (which could correspond to the onset of yielding or other processes such as softening).^13^ We refer to the strain range between *γ*_nl_ and *γ*_f_ (flow strain) as the ‘flow transition’ region. *γ*_0_ > *γ*_f_ marks the start of the ‘bulk flow’, indicating the response is liquid-like, with *G*′′ > *G*′. The distinction of these three regions is shown in Fig. 6(c) for Gr – 30 wt%. The flow strains (*γ*_f_) remain very close for all the samples, suggesting that although the formulations with high Gr loading start to change ‘early’, they enter the bulk flow (liquid-like) phase at similar strain values to other formulations (Fig. 6(a)). Unlike *σ*_nl_, the flow stress (*σ*_f_) shows an increasing trend with Gr loading in the formulation (Fig. 6(b)). This suggests that although a relatively similar stress is needed to soften the material, a higher stress is needed to achieve ‘bulk flow’ as the Gr concentration increases. The ‘flow transition’ region is quantified using the ratio of *σ*_nl_ and *σ*_f_ (given by the inverse of the flow transition index, FTI^−1^),^17^ with a FTI^−1^ = *σ*_nl_/*σ*_f_ value of around 0.6 for Gr – 10 wt%. However for the sample with the highest Gr loading (Gr – 50 wt%) *σ*_nl_/*σ*_f_ drops to less than 0.1 (Fig. 6(a), right *y*-axis), resulting in a more extensive ‘flow transition’ region (Fig. 6(b)). This shows that any modification of formulation parameters can change the solid-to-liquid transition, which can negatively impact on printing performance. Here, the addition of Gr up to 50 wt% results in a loss of resolution and worse shape fidelity (Fig. 3).

#### 3.3.2 Shifts in ‘flow transition’ and yielding mechanisms

**Fig. 7:**
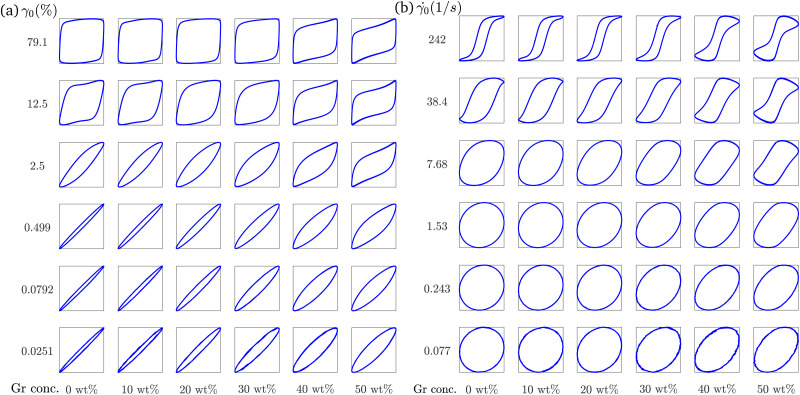
(a) Elastic LB curves (*σ*/*σ*_0_*vs. γ*/*γ*_0_), (b) Viscous LB curves (*σ*/*σ*_0_*vs. γ̇*/*γ̇*_0_) of Gr-F127 formulations with Gr concentration varying from 0 wt% to 50 wt% (left to right). The strain and strain rate amplitudes are increasing from bottom to top.

**Fig. 8:**
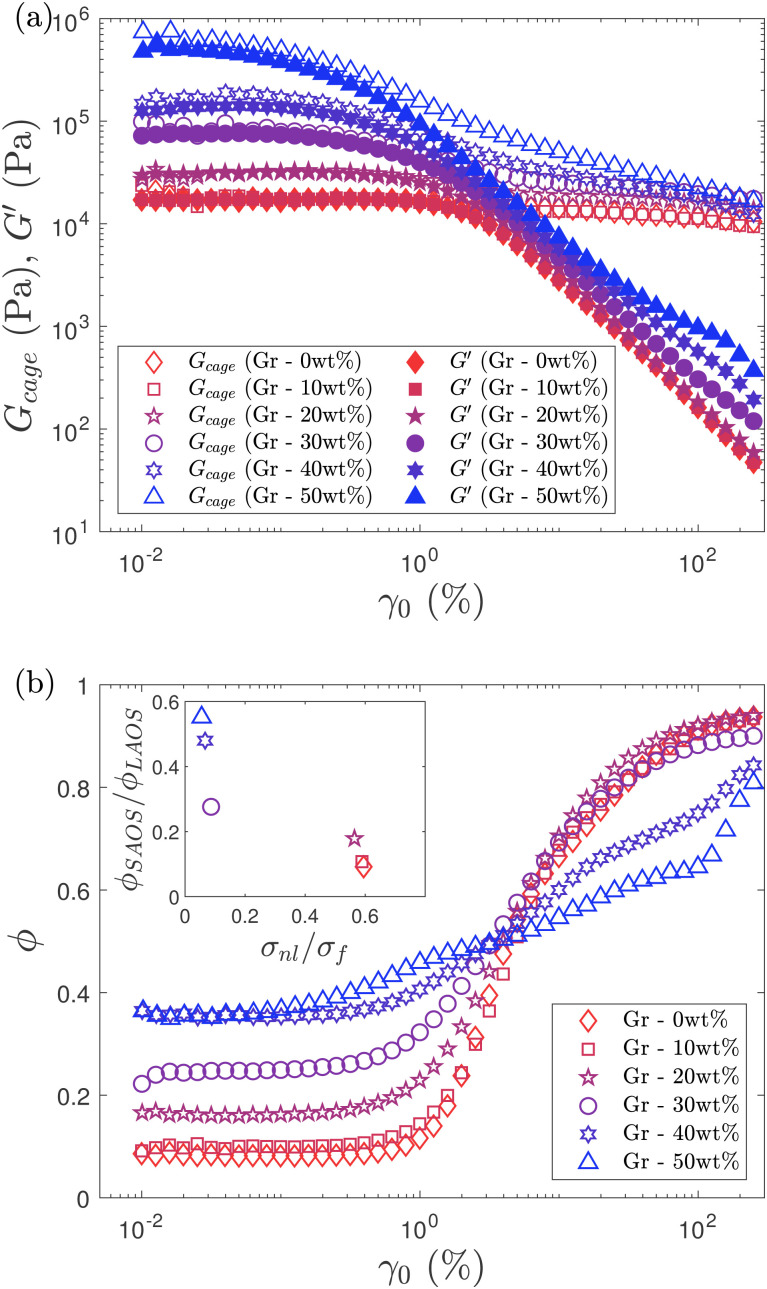
(a) Evolution of *G*_cage_ and *G*′ with strain amplitude, *γ*_0_. All the formulations show a very similar trend for *G*′ regardless of Gr content. However, F127 and formulations with Gr loading below 20 wt% show a very small variation in *G*_cage_, which almost remains constant at any *γ*_0_ value. As Gr concentration increases there is a considerable decrease of *G*_cage_ as *γ*_0_ increases. (b) Variation of the plastic dissipation ratio*, ϕ* with strain amplitude*, γ*_0_ for different formulations. Inset on top left of figure (b) shows the ‘Flowability’ map based on dimensionless ratios calculated from linear metrics (*ϕ*_SAOS_/*ϕ*_LAOS_) plotted *vs.* yield to flow stress ratio (*σ*_nl_/*σ*_f_, or FTI^−1^)*. ϕ*_SAOS_ values were obtained at *γ*_0_ = 0.02% and *ϕ*_LAOS_ at *γ*_0_ = 100%. Formulations that produce parts with good resolution fall within the right-bottom region. This map shows a trend shifting from this area towards top-left as the resolution worsens with increasing Gr content.

**Fig. 9:**
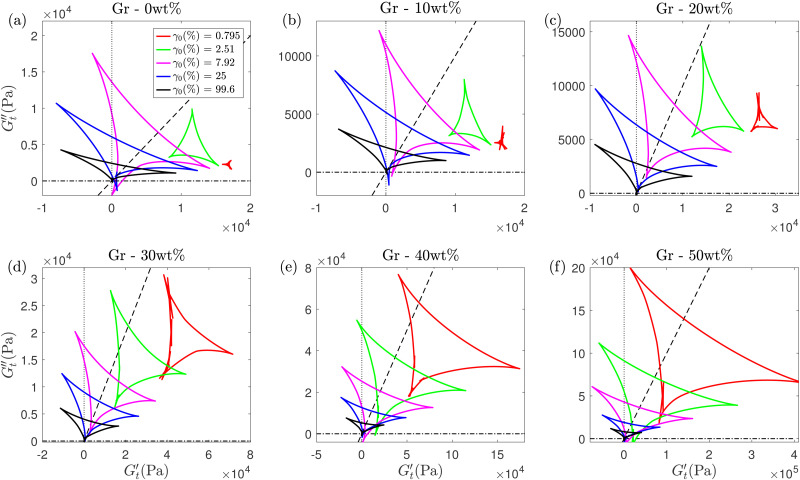
SPP analysis of the inter-cycle rheological transition at various strain amplitudes using Cole–Cole plot for Gr concentrations of (a) 0 wt%, (b) 10 wt%, (c) 20 wt%, (d) 30 wt%, (e) 40 wt% and (f) 50 wt%. The dashed lines represent 
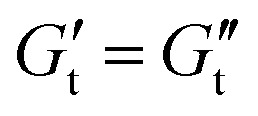
. The line colors in (b)–(f) show the data for the same strain amplitudes as in (a).

The quantitative differences discussed in Section 3.3.1 suggest that not all the yielding processes are the same for the formulations in this work. LB curves can provide a qualitative visual description of the response of a material during an amplitude sweep. Although it is not possible to quantify the recoverable and unrecoverable processes from our experiments and analysis, the LB curves can be used to calculate *G*_cage_ (Section 2.3.3). These LB plots are a helpful reference when we analyze the solid-to-liquid (‘flow transition’) region using *ϕ*, *G*_cage_, and the SPP framework. For formulations with good printing resolution at Gr concentrations below 20 wt% and at small strain amplitudes (*γ*_0_ < 0.5%), the elastic LB projections in Fig. 7(a) are close to a straight line and the viscous LB projections in Fig. 7(b) are close to a circle, indicating the dominance of elastic contributions. However, as the Gr concentrations is increased above 20 wt%, the curves in Fig. 7(a) evolve to ellipsoidal shapes with increasing areas. This indicates that as the Gr concentration increases, there is a greater energy dissipation even at very low strain values. This behaviour is further quantified using *ϕ* trends and the SPP analysis (Fig. 8(b) and 9). As the amplitude increases, the elastic and viscous LB curves get distorted at medium and large amplitudes. For low Gr concentrations, at the largest strain amplitude, the elastic LB curves are close to a square shape (Fig. 7), which corresponds to nearly perfect plastic flow.^53^

Given the significant distortions in the LB plots at large strain amplitudes, qualitative observations are not sufficient to describe the instantaneous rheological state of the material. Two different parameters (the apparent cage modulus (*G*_cage_) and the plastic dissipation ratio (*ϕ*)) are calculated to quantify the information provided by the LB curves and support our physical interpretations.

#### Apparent cage modulus

The loss modulus (which has been described as a composite parameter)^71^ shows distinctive trends in the flow transition region: the *G*′′ overshoot disappears with increasing Gr content. It is not possible to differentiate the recoverable and unrecoverable processes that the formulations might undergo, and which we believe are playing an important role. However, we can gain some insight from the analysis of the *G*_cage_ (eqn (2)) evolution. This is a parameter specifically defined for yield stress fluids^41^ that represent the cage elasticity (or average energy stored elastically normalised by the recoverable strain amplitude). *G*_cage_ shows a different trend depending on the Gr content (Fig. 8(a)). *G*_cage_ matches the storage modulus at small strain amplitudes. For formulations with less than 20 wt% Gr, *G*_cage_ remains constant while *G*′ decreases as the strain amplitude increases. However, when the Gr concentrations increase above 20 wt% and up to 50 wt%, *G*_cage_ also decreases with increasing strain amplitude. The higher the content of Gr powder, the more dramatic is the drop in cage elasticity with increasing strain amplitude (Fig. 8(a)). These results suggest that in the absence or with low concentrations of Gr (<20 wt%), the F127 hydrogel network is determining the yielding mechanism. F127 micelles or “cages” (that arrange in a cubic structure)^72,73^ are able to deform elastically until they reach a point when they might break and evolve from purely elastic behaviour to perfect plastic flow. However, when the Gr concentration increases (>20 wt%), the F127-Gr microstructure or network changes, it seems unable to evolve in the same way. The results evidence that the F127-Gr “cages” do not only deform elastically, that they start to soften, rearrange or perhaps break early on in the flow transition region leading to an increase of the dissipated energy (evidenced by trends in *ϕ* values and SPP results). This translates in a decrease of the apparent cage modulus over two orders of magnitude for the highest Gr concentration (Fig. 8(a) and (b)). The cage elasticity seems to collapse and even out for all formulations at large amplitudes, which based on its definition (average energy stored elastically normalised by the recoverable strain amplitude^41^) suggests that: (i) the F127 network in the absence of Gr undergoes recoverable processes and (ii) as the Gr concentration increases, there is an increase of unrecoverable processes taking place.

#### Dissipation ratio

The trends in *ϕ*^13,53^ values are distinctive for each formulation (Fig. 8(b)). At small strain amplitudes, formulations with low Gr content (<20 wt%) undergo the least energy dissipation with *ϕ* values below 0.1 (Fig. 8(b)). As Gr concentration increases, *ϕ* shows a near-monotonic increasing trend for small *γ*_0_ which is consistent with the expanding area of the elastic LB curves (Fig. 7(a)); energy is being dissipated even at very small strain values. As we move towards the ‘flow transition’ region, *ϕ* increases for all the formulations with a clear trend that depends on the Gr content. For low concentrations (<20 wt%), *ϕ* values show a steep change at intermediate strains (from *ϕ* ≈ 0.1 to *ϕ* ≈ 0.9, Fig. 8); the energy dissipation takes place in a narrow strain space (that corresponds to the ‘flow transition’ region). However, when Gr loading increases up to 50 wt%, *ϕ* values vary between ∼0.35 and ∼0.8, showing a gradual increase in a broader strain range, and at a considerably smaller rate as strain increases (Fig. 8(b)). Energy is being dissipated from the start and throughout the amplitude sweep. The higher the Gr loading, the lesser is the increase in *ϕ* as we move from SAOS to LAOS regions. *ϕ* values for all the formulations cross each other at around *γ*_0_ ≈ 4%. In the LAOS region, the low Gr formulations approach the perfect plastic response which is also consistent with the near square shape of the elastic LB curves (Fig. 7(a)) for large *γ*_0_.

The F127 hydrogel (without Gr) shows a ‘clean’ transition from viscoelastic solid (with an almost purely elastic response) to a nearly perfect plastic flow (almost square shape in LB curve) in a narrow strain (and stress) region. The F127 microstructure seems able to store energy up to higher strains, as evidenced by sustained cage elasticity values. While the formulations with high Gr concentrations (above 30 wt% Gr) that lead to worse print quality undergo continuos and gradual energy dissipation at all strain values. These samples exhibit a narrow (almost inexistent) LVR, an extensive “flow transition” region, and a considerable drop of the cage elasticity at large amplitudes.

These results support our hypothesis on the impact of Gr concentration in microstructure, yielding mechanism and energy transitions, that are playing a role in the loss of printing resolution (discussed in Section 3.1). Linking the observed and quantified trends in *ϕ*, *G*_cage_ values, and printing resolution (loss of porosity between filaments, Section 3.1), it suggests that the formulations with least energy dissipation in an extensive LVR, and a narrow ‘flow transition’ (potentially undergoing recoverable processes) lead to good replicas of the pre-designed features.

Based on these insights, we propose a flowability map using dimensionless relationships calculated from linear measures: *σ*_nl_/*σ*_f_ (or inverse of flow transition index,^17^ FTI^−1^) and *ϕ*_SAOS_/*ϕ*_LAOS_ (inset of Fig. 8(b)). Values of *ϕ*_SAOS_/*ϕ*_LAOS_ ≈ 0 and FTI^−1^ ≈ 1 (bottom-right side of the map, below 20 wt% Gr) correspond to a ‘clean’ transition from viscoelastic solid to perfect plastic flow, and good printing resolution. While values of *ϕ*_SAOS_/*ϕ*_LAOS_ ≈ 1 and FTI^−1^ ≈ 0 (above 40 wt% Gr) are associated to a narrow LVR, and an extensive solid-to-liquid (‘flow transition’) region with increased and continuous energy dissipation (which might not be fully recoverable). This map enables to compare the flow transition region to analyse trends and establish links with printing resolution. In order to determine and quantify the recoverable and unrecoverable processes, a different framework will be applied in future work.^71,74^

#### 3.3.3 Microstructure evolution during yielding

The SPP framework enables us to study the microstructure evolution during the solid-to-liquid transitions by probing the intra-cycle response of the formulations as they change during an oscillation cycle. Cole–Cole plots at a specific strain amplitude provide insights into the intra-cycle rheological transitions, and the variations in area and location of trajectories at increasing strain amplitudes provide information on inter-cycle rheological transitions.^37,57^ For example, if the deltoid has a bigger area, there is a wider range of structural rearrangements within the material at that strain amplitude.^56^ In the LVR, the Cole–Cole plots are expected to have a very small area as the structures are in an equilibrium state.^75^ The evolution of the deltoid's size and orientation with increasing *γ*_0_ for all the formulations (Fig. 9) shows clear trends that quantify the different yielding transitions depending on the Gr content. For low Gr content (≤20 wt% Gr), the area of the deltoids remains very small at low *γ*_0_ values, and then increases until around *γ*_0_ ≈ 8% where it reaches a maximum value. This strain amplitude is very close to the flow strain (*γ*_f_) values for these formulations (Fig. 6(a)). The increase in area is due to the samples’ microstructures leaving the LVR, undergoing rearrangements and reaching a maximum in energy dissipation (largest area at ≈*γ*_f_). After this maximum (at strain amplitudes above *γ*_0_ ≈ 8%) the rearrangements and energy dissipations ease off (smaller deltoids) with increasing *γ*_0_ due to the break-down of the microstructural networks as strain amplitude increases further into LAOS, leading to weakening of the nonlinear response.^75^ The Cole–Cole inter-cycle plots evidence the shift in the solid-to-liquid (‘flow’) transition and the trend of structural rearrangements that the formulations with high Gr concentrations (40 wt% and 50 wt%) undergo. For these, the deltoids have the largest area at smaller strain amplitude values (Fig. 9(e) and (f)). At *γ*_0_ = 0.795%, the formulation with the highest Gr content is undergoing a wide range of structural rearrangement at very small strains (Fig. 9). The area of the deltoids decrease almost monotonically with increasing strain amplitudes (unlike formulations with low Gr loading with a maximum in deltoid's area at ≈*γ*_f_), evidencing a continuous energy dissipation and weakening of the microstructure almost from the start of the amplitude sweep. These plots illustrate the impact of increasing Gr loading and how the trajectory evolution shifts at around 30 wt%. This is a critical concentration above which the ‘cages’ seem to loose their elasticity (3.3.2), perhaps due to the presence of Gr particles modifying the micelle arrangement in a cubic phase.^72,73^ The Cole–Cole plots reinforce the findings discussed in Section 3.3.1; they provide complementary evidence to better understand the underlying physical processes responsible for the loss of printing resolution.

### 3.4 ‘Recoverability’ or ‘ability to rebuild’

A deeper understanding of the yielding phenomenon and its physical processes paves the way to establish new links between rheological metrics, print quality, and shape fidelity. The findings based on *ϕ*, *G*_cage_, and Cole–Cole plots show that changing one ingredient in the formulation leads to a shift in the ‘flow transition’ and the physical processes the microstructures undergo, which might compromise their recovery. Here, the term ‘recovery’ is used in the DIW context which refers to the transition kinetics and the extent of rebuild during printing, *i.e.* from a fluid-like flow behaviour during extrusion to elastic shape retention once extruded.^14,17,42,76^ This should not be confused with the definition of recovery in the yield stress fluids field, in which recoverable strain is associated to elastic processes while unrecoverable strain is due to plastic or viscous processes.^74^ The calculation of the recoverable and unrecoverable strains can be carried out using the recently developed ‘recovery rheology’ framework by Rogers and co-workers.^54,74,77^ In this work, we have not quantified the recoverable and unrecoverable strains, and remains the subject of our future research.

**Fig. 10:**
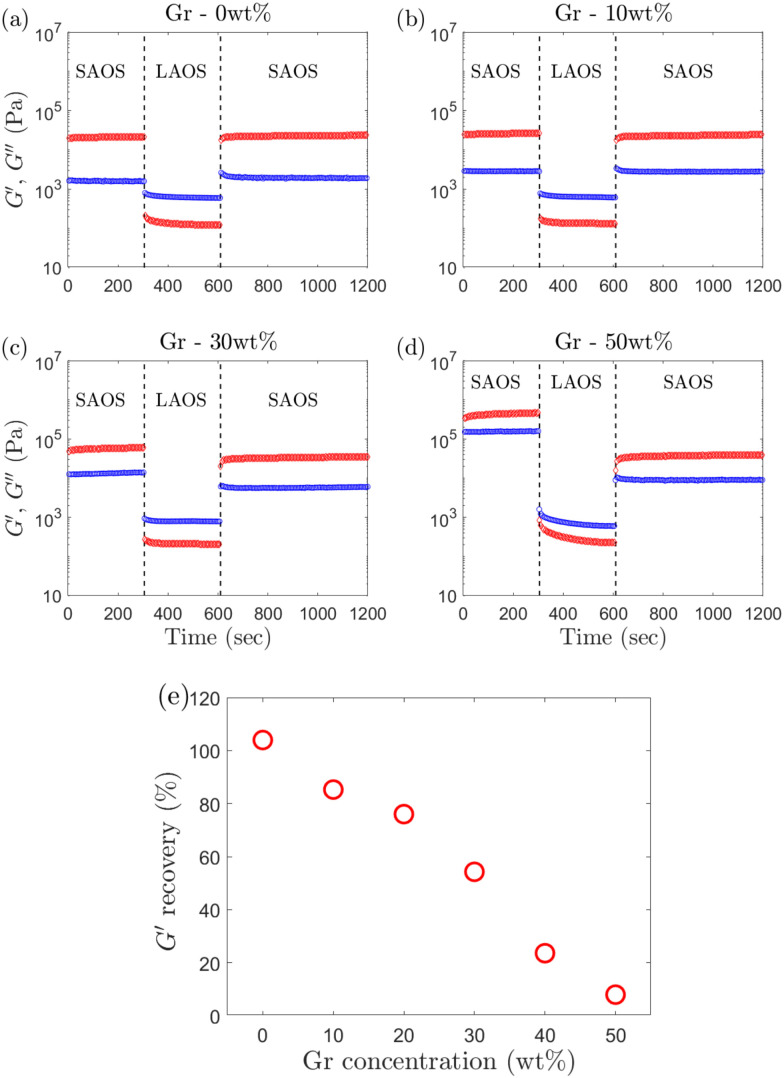
Recovery of *G*′ (red symbol) and *G*′′ (blue symbol) with varying Gr concentrations, (a) 0 wt%, (b) 10 wt%, (c) 30 wt%, and (d) 50 wt%. (e) Recovery of *G*′ after the LAOS step with varying Gr concentrations. The *G*′ value, 200 seconds after the start of recovery (called as 
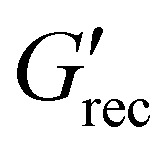
), is considered for the calculation, in order to avoid any major transient phenomenon (which exists until around ≈100 seconds as can be seen in Fig. 11(a)).

Quantification of the extent and the timescales of the structural recovery post-yielding (when leaving the nozzle tip) is crucial to elucidate the impact on the recoverability criteria that is linked to print quality in DIW.^14^ Here we establish rheological metrics to quantify ‘recoverability’ (in terms of extent and timescales) using a 3-step recovery test (Section 2.3.5). The extent of recovery is calculated as 
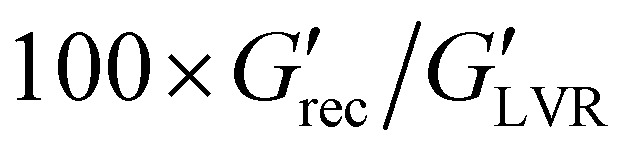
, where 
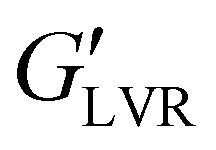
 is the stiffness before the LAOS step and 
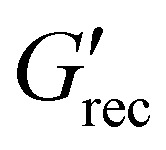
 is the plateau stiffness values after the LAOS step (Fig. 10).

**Fig. 11:**
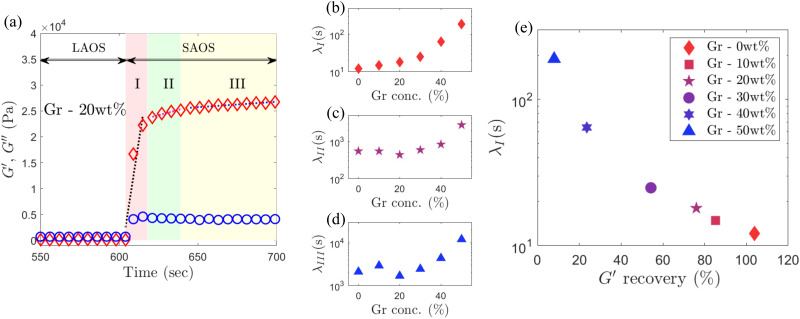
(a) Recovery curve highlighting the time evolution of the *G*′ (red symbol) and *G*′′ (blue symbol) in the LAOS to SAOS recovery region of 20 wt% Gr suspension. The recovery region is separated into three different stages: I, II and III. The dotted line in each region is used to calculate the gradient, d*G*′(*t*)/d*t* for the mutation time calculation (eqn (7)). (b)–(d) Mutation time variation with Gr concentrations for stages (b) I, (c) II and (d) III, calculated using eqn (7). (e) ‘Recoverability’ map using two key rheological parameters: mutation time for the stage I (*λ*_I_) and the percentage recovery of *G*′.

**Fig. 12:**
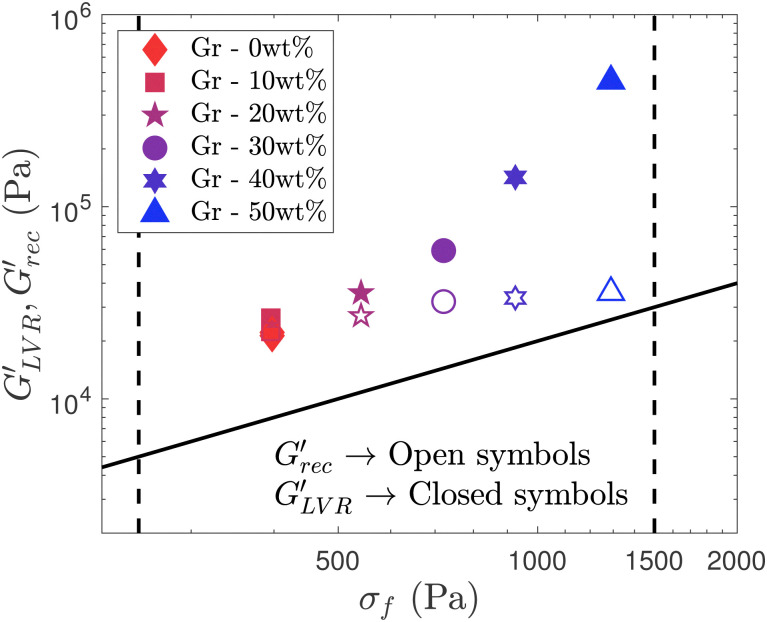
Material strength map (storage modulus *vs.* flow stress (*σ*_f_)) comparing 
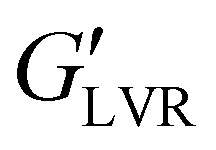
 (filled symbols) and 
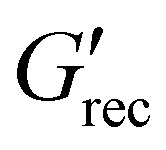
 (open symbols) after the recovery stage in 3-step recovery experiments. The two vertical lines for *σ*_f_ limits, *σ*_f_ = 250 Pa and *σ*_f_ = 1500 Pa, and the diagonal line that represents the figure of merit (
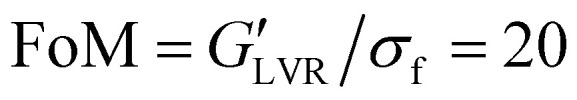
). The increase of Gr content leads to an increase of the storage modulus in the LVR, however the stiffness after the LAOS step remains in the same order of magnitude for all the formulations.

It could be argued that the recovery test does not replicate the actual conditions during the DIW process and that other factors should be considered, such as precise shear conditions and the differences in stress distribution within the tip compared to the gap within the plate geometries. The main limitation is that the precise shear stress/strain that the material experiences during the extrusion is uncertain. Due to the nature of these complex fluids, the true shear stress/strain conditions are not only process (*e.g.* extrusion rate, tip diameter, length and shape and printing speed) but also material dependent. A more sophisticated approach is needed to provide a better prediction of the shearing conditions during the DIW process. For example, carrying out a computational fluid dynamics (CFD) study of ink flow during the extrusion process with realistic boundary conditions and models for the inks, and monitoring the evolution of moduli in time and space would address this gap. A CFD study for (time-dependent) yield stress fluids is out of the scope of this work but we will strengthen this area of research in the future. Although the recovery test admittedly has some limitations to accurately quantify the shear conditions and therefore the precise extent of the recovery taking place in the DIW process, we can still confidently use it to compare their recoverability performance as one of the printability criteria for any formulation. This is because applying a large amplitude (*γ*_0_ = 100%) oscillation for 300 s ensures that the samples are subjected to extreme shear conditions. Thus any material able to recover and achieve certain structural (*G*′) values under these conditions (Fig. 12), it should be able to perform during the DIW process, as long as the three printability criteria are all met (flowability, recoverability, and material strength).

The extent of the recovery is important to ensure the material strength is enough to create free-standing structures. The kinetics or timescales of the recovery are critical to ensure that this is achieved within the DIW process. Here we propose metrics to quantify the recoverability timescales through the evolution of *G*′ and *G*′′ values with time in the LAOS to SAOS recovery region for our representative set of formulations (Fig. 11(a)). The recovery transition can be divided into three regions/stages: a steep and sharp initial recovery of *G*′ (stage I), followed by a region with slower increase (stage II), and finally the values reach a plateau (stage III).^17^ The mutation times, *λ* values (eqn (7)), are calculated for all formulations at each of the three stages (Fig. 11(b)–(d)). The magnitude and timescale of the structural recovery in stage I are key in the short timescales of the deposition process in DIW,^17^ with values of *λ*_I_ ≈ 10 s for ‘printable’ graphene oxide (GO) formulations. We see here that *λ*_I_ is close to 10 s for F127 (0 wt% Gr) matching the values of GO suspensions.^17^ As the Gr concentration increases up to the threshold value at which the yielding mechanism change (30 wt% Gr, Section 3.3), *λ*_I_ increases slowly up to ≈25 s, but it then shows a steep increase reaching around *λ*_I_ ≈ 200 s at the highest solid loading (50 wt% Gr). *λ*_II_ and *λ*_III_ remain almost independent of the Gr concentrations until around 20–30 wt% Gr, and after that, they both increase rather steeply (Fig. 11(c) and (d)). The trends in mutation times for Gr formulations match those observed in Section 3.3, thus highlighting again a shift in behaviour at around 30 wt% Gr. Above which, the timescales of the structural recovery in stage I are not fast enough (likely due to the different nature of the physical processes that the microstructures undergo, Section 3.3.1) to recover enough stiffness in time to retain filament shape, which leads to worse shape fidelity overall as shown in Section 3.1 (Fig. 3).

Selecting two distinctive metrics from the recovery tests, *λ*_I_ (*s*, *y*-axis) and extent of the structural recovery, 
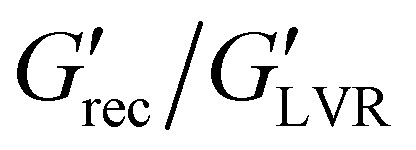
 (%, *x*-axis), we build a recoverability map (Fig. 11(e)). This map shows that the timescales (*λ*_I_ values) and the extent of the *G*′ recovery are directly correlated (Fig. 11) and that both depend on Gr concentration. There is a clear trend with Gr loading: at low Gr content, the *λ*_I_ values are small (≈10 s) and the stiffness recovers almost fully (≈100%); but as Gr concentration increases up to 50 wt%, *λ*_I_ increases up to ≈200 s while the recovery drops dramatically. This trend is different to that observed for GO suspensions,^17^ which showed a consistent increase in both stiffness and gradients leading to fairly similar *λ*_I_ values at different flake concentration. This is because the mechanism of network formation are different between GO and Gr-F127. For GO, the large two dimensional sheets are able to establish flake–flake interactions that can break down and rebuild.^17,62^ However, the increase of Gr concentration above the threshold value leads to a loss of the F127 hydrogel matrix and the Gr particles are not able to establish a network that can deform elastically. The recoverability Ashby-type chart provides new insights to quantify characteristic trends that correlate with the observed evolution in print quality.

### 3.5 ‘Material strength’

Two measures of materials strength commonly used in DIW,^12,14,17,78^
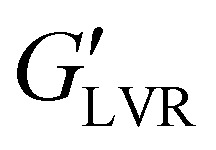
 and *σ*_f_ can be plotted in an Ashby-type chart^13^ to create a ‘printability map’, for different yield stress fluids used in bioprinting and energy applications (Fig. 12).^13^ This map also includes dashed vertical lines delimiting the region of interest, or printability window, including: two vertical lines for *σ*_f_ limits, *σ*_f_ = 250 Pa and *σ*_f_ = 1500 Pa, and a diagonal line that represents the figure of merit (
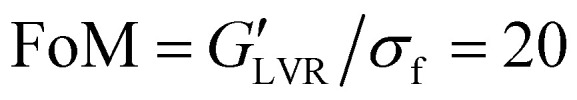
).^78^ These limits are used as guidance based on data from existing formulations, but they need to be used in the application context. The left-bottom region is associated with the ‘softer’ formulations suitable for 3D printing of parts with limited complexity. The right-top region is associated with ‘stiff’ formulations that provide better shape retention and enable to create structures with intricate features.^13^ All the formulations in this work (Fig. 12) fall within the ‘printable’ region. As the Gr concentration in the formulation increases, both 
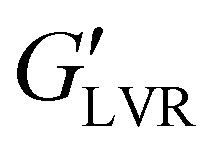
 and *σ*_f_ increase, thus moving towards the top-right region in this map (Fig. 12). Here, we show that these two parameters may not be sufficient rheological metrics to describe the behaviours linked to printability.

From the recovery test, we see that the extent of the recovery considerably drops from almost 100% for F127 (or 0 wt% Gr) to less than 10% at the highest solid loading (50 wt% Gr) (Fig. 10(e)). The microstructures after the LAOS step are not able to reform or store the same amount energy elastically (considerable drop of *G*′ values) within the timescale of the experiment (*T*_SAOS,2_ = 600 seconds). F127-based formulations with varying ceramic contents have also shown similar results in 3ITT.^12^ The *G*′ value dropped around 3% for pure hydrogel and 94% for 70 wt% ceramic loading.^12^ Although the increase in solid loading leads to a considerable loss of elasticity (*G*′↓), the formulations might still be ‘printable’ albeit resulting in poorer shape fidelity and print quality. This is because the material only needs certain values of *G*′ to retain the filament shape and support the printed structure without slumping or collapsing. Different formulations can be compared in the material strength map by plotting both, 
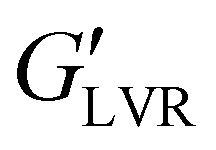
 and 
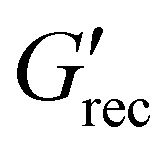
 (Fig. 12). The results from the recovery experiment show that the stiffness values (
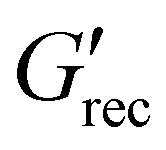
) for all the formulations remain at the same order of magnitude as for the F127 formulation base (interestingly, 
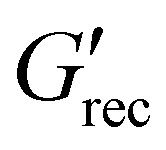
 value level off similarly to ≈*G*_cage_ values), Fig. 8; 
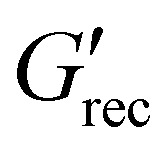
 only slightly increases with Gr content. All the formulations fall within the “printable” limits regardless of the stiffness (*G*′) values considered in the map (Fig. 12). However, when we focus on the storage modulus from the recovery test (
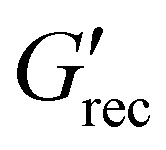
), the values in the map do get closer to the FoM diagonal^13,78^ as Gr increases, which translates in a reduction of print quality or shape fidelity with increasing Gr content.

### 3.6 Rheological descriptors and maps for DIW formulations

**Fig. 13:**
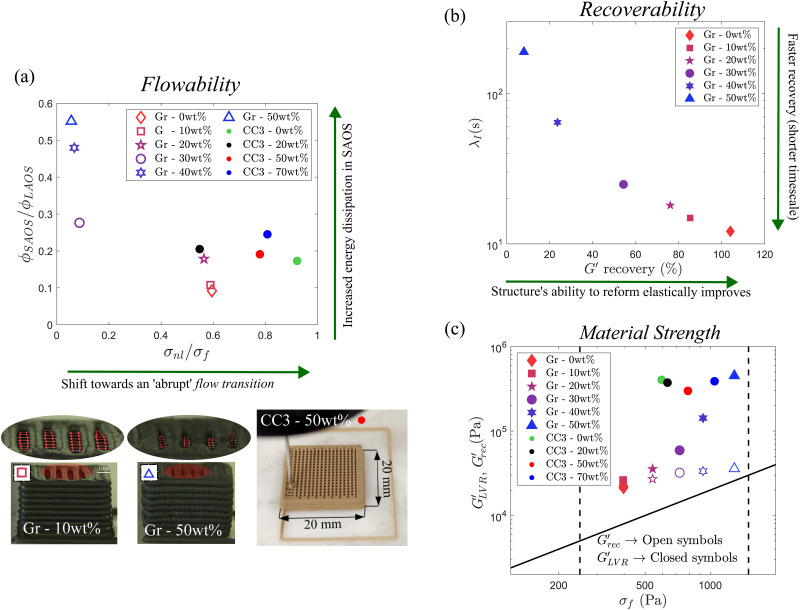
Printability maps based on three rheological criteria: (a) flowability, (b) recoverability and (c) material strength. The formulations with *ϕ*_SAOS_/*ϕ*_LAOS_ → 0 and *σ*_nl_/*σ*_f_ → 1 (shown using arrow in figure (a)) in the flowability map, and *λ*_I_ → 10 s and 
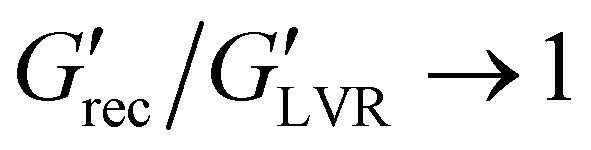
 (shown using arrow in figure (b)) in the recoverability map produce printed structures with the highest resolution. In case of limited and/or slow recovery of the formulations, 
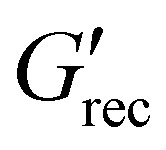
 provides a more accurate assessment of material strength than 
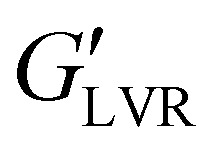
. Data from our recent work^79^ for 3D-printed porous organic cages (CC3) has been added in these maps. Four different composition of formulated inks with varying CC3 loading (0 wt%, 20 wt%, 50 wt% and 70 wt%) were used for the study. The formulation with 50 wt% of CC3 displayed the best printing behaviour in good agreement with the analysis of the LAOS results.^79^ Images of representative structures (bottom-left) printed using Gr – 10 wt% and Gr – 50 wt% formulations, (taken from Fig. 3(c) and (i), respectively), and a printed structure made with CC3-50 wt% showing good printability and shape fidelity.^79^

The findings from this work provide a framework to quantify three rheological criteria that are linked to the printability^14^ of soft materials for DIW. We have identified rheological metrics able to quantify the distinctive behaviours observed during *in situ* DIW monitoring for a set of representative model formulations. These metrics are compiled in three Ashby-type maps. Each map focuses in one of the three rheological parameters that define printability: flowability, recoverability and material strength.^14^ The three maps (Fig. 13) show consistent trends for all the formulations in this study providing new insights into the links between rheology and printing resolution.

The flowability map (*ϕ*_SAOS_/*ϕ*_LAOS_) *vs. σ*_nl_/*σ*_f_ (also referred to as FTI^−1^) provides a comparison of the materials’ ‘flow transition’ (Fig. 13(a)). The *y*-axis of this map is related to the underlying physical process in microstructure rearrangements: through the ratio *ϕ*_SAOS_/*ϕ*_LAOS_ that compares the evolution of the energy dissipation between the LVR and the “bulk flow” regions (Section 3.3.1). The *x*-axis is linked to the extension of the ‘flow transition’ region (FTI,^17^ Section 3.3.1). The formulations that lead to better printing resolution (bottom-right region, Fig. 13(a)) exhibit *ϕ*_SAOS_/*ϕ*_LAOS_ values below 0.2 and *σ*_nl_/*σ*_f_ values close to 1. Small *ϕ*_SAOS_/*ϕ*_LAOS_ values are associated with minimal energy dissipation at small amplitudes (SAOS), and high energy dissipation at large amplitudes (LAOS); *σ*_nl_/*σ*_f_ values close to 1 represent an “abrupt” or narrow ‘flow transition’ region. When *ϕ*_SAOS_/*ϕ*_LAOS_ > 0.2 and *σ*_nl_/*σ*_f_ values approach to 0, print quality worsens (top-left region, Fig. 13(a)). The results (explained in more detail in Section 3.3.1) suggest that the loss of printing resolution can be due to changes in the physical processes the samples undergo when they yield (*e.g.* energy being stored or dissipated to different levels) and the widening of the flow transition region (*σ*_nl_/*σ*_f_ → 0).

The recoverability map correlates the timescales and extent of the recovery by plotting *λ*_I_*vs.* % of *G*′ recovered (Fig. 13(b)). Formulations that fall in the bottom right region correspond to better shape fidelity (low Gr loading) (Section 3.1). Those that fall towards the top left of the map, do not perform well during DIW due to limited and slow structural recovery. The top-left region in this map is associated with poor recoverability.

The material strength map (Fig. 13(c)) that we propose, includes two different measures of strength: the elastic modulus in the LVR before the LAOS step, 
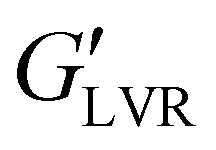
, and the elastic modulus in the plateau region in stage III from a three step recovery experiment, 
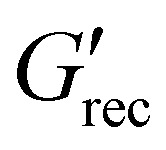
 (Sections 2.3.5 and 3.4). Including these two metrics accounts for potential recoverability issues. The map (Fig. 13(c)) clearly shows that 
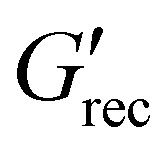
 values diverge as the Gr content increases due to the damage of the structure, which is unable to regain its initial mechanical strength within the DIW timescales. We recommend to not base the material strength printability criteria on 
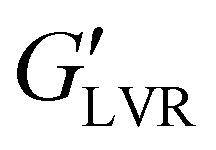
 values alone, unless it is known that the formulations of interest are able to yield and reform in the required timescales. Instead, considering 
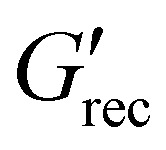
 can provide a more accurate assessment of material strength. The maps (Fig. 13) also include data from optimised (printable and functional) formulations for gas filtration.^79^ These hybrid mixtures have different composition and microstructure, formed by bentonite clay, porous organic cages (not to be confused with the concept of cage elasticity) combined with a much smaller amount of F127.^79^ This showcase that samples with very different microstructures that are printable with good resolution, do fall in the expected regions in the flowability map as the graphite-formulations.

We recommend combining these maps to consider the interplay between yielding, recovery and strength holistically. The results convey that formulations with *ϕ*_SAOS_/*ϕ*_LAOS_ → 0 and *σ*_nl_/*σ*_f_ → 1 in the flowability, and *λ*_I_ → 10 s and 
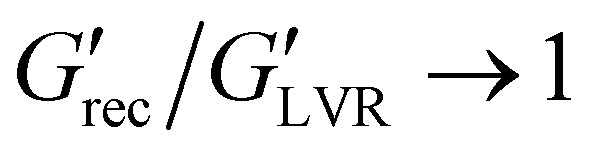
 in the recoverability maps produce printed structures with the highest resolution. In case of limited and/or slow recovery of the formulations, 
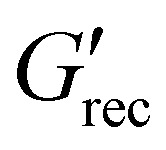
 provides a more accurate assessment of material strength than 
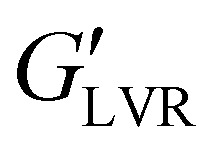
.

## 4 Conclusions

With the rapid advancement made in the field of DIW, linking rheology and printability of yield stress fluids has become a very active area of research. There are several rheological parameters (linked to three printability criteria previously reported: material strength, flowability and recoverability) of formulations that play a role in printing desired 3D structures with accuracy. Gr based inks are gaining popularity in the DIW community for their diverse range of applications, such as in stretchable strain sensors, embedded printing and as fugitive inks. Combining Gr powders and pluronic F127 hydrogel we have formulated a set of samples that exhibit typical behaviours in DIW. The formulations here studied are printable (they steadily flow and can be deposited to create a free-standing 3D structure), however they produce parts with different resolution. Using strain amplitude sweeps and recovery experiments, combined with *in situ* monitoring, we have identified rheological metrics that show consistent trends with printing resolution.

*In situ* monitoring enables to assess the accuracy of the printing process through the quantification of structural features such as the pore area or gap distance between filaments. We have found that the physical processes and energy transitions taking place during the yielding phenomenon play a central role in the performance of DIW formulations. We have not found a single metric or parameter that can be used to define “printability”, because there is more than one criteria that should be met.

We have found complementary metrics that show distinctive trends evidencing the interplay between yielding, recovery and strength and how they correlate with printing performance. Based on our findings, we propose three maps to quantify the flowability, recoverability and strength of DIW formulations, which bring together the three printability criteria.

These maps show consistent trends for the representative set of formulations studied. Combining rheology experiments with *in situ* monitoring, this work provides a guideline to characterise yield stress fluids for DIW. The systematic analysis of rheological metrics compiled in three Ashby-type maps, and the new insights into the underlying physical processes that take place during the yielding of different formulations, pave the way to establish links between rheology, printability and printing resolution.

## Author contributions

Conceptualization (EGT). Data curation (RA). Formal analysis (RA, EGT). Funding acquisition (EGT). Investigation (EGT, RA). Methodology (RA, EGT). Project administration (EGT). Resources (EGT). Software (EGT, RA, Freeware package by Prof. Simon Rogers). Supervision (EGT). Writing original draft (RA, EGT). Writing review and editing (EGT).

## Data availability

Some of the data (videos) supporting this article have been included as part of the ESI.† The raw data for the rheological measurements and the analysis can be made available upon acceptance and request. The FT analysis has been made using our own MATLAB script, that can be made available upon acceptance and request. The SPP analysis has been made using MATLAB functions provided by Simon Rogers (SPP Freeware package) as stated in the manuscript.

## Conflicts of interest

There are no conflicts to declare.

## Supplementary Material

SM-020-D4SM00758A-s001

SM-020-D4SM00758A-s002

SM-020-D4SM00758A-s003
